# Evolution of EEG Findings in Patients with Acute Brain Injury

**DOI:** 10.21203/rs.3.rs-4649424/v1

**Published:** 2024-10-14

**Authors:** Jackson A Narrett, MarieElena Byrnes, Emily J Gilmore, Lawrence J Hirsch, Vineet Punia, Adithya Sivaraju

**Affiliations:** Yale New Haven Hospital; Cleveland Clinic Health System: Cleveland Clinic; Yale New Haven Hospital; Yale New Haven Hospital; Cleveland Clinic Health System: Cleveland Clinic; Yale New Haven Hospital

**Keywords:** Acute symptomatic seizures, EEG, epileptic

## Abstract

**Introduction:**

Increasing use of continuous EEG monitoring (cEEG) provides the opportunity to observe temporal trends in EEG patterns during the acute phase of brain injury. These trends have not been extensively documented.

**Methods:**

We conducted a retrospective chart review of patients undergoing cEEG between January 1st and June 30th, 2019, at two academic medical centers. Only patients with acute brain injury having electrographic or electroclinical seizures or epileptic EEG findings on day one of monitoring and ≥ two calendar days of cEEG were included. The temporal evolution of EEG patterns was depicted as a heatmap.

**Results:**

Of 1356 screened patients, 101 met the study criteria. Clinical acute symptomatic seizures occurred in 30 patients (29.7%) prior to EEG. The median number of days of cEEG was four (IQR 3–6). Amongst patients with electrographic seizures, status epilepticus, generalized periodic discharges, or sporadic epileptiform discharges, 24.6% had improvement and 54.1% had resolution of epileptic EEG findings by the final day of monitoring. In contrast, 65% with lateralized periodic discharges or lateralized rhythmic delta activity persisted or worsened. Overall, 61.4% (62/101) showed either improvement (19.8%) or resolution (41.6%) of their EEG findings prior to hospital discharge. Of the 36 patients with follow-up EEGs at a median of 4.5 (IQR 3–8) months after admission for acute brain injury, 83% (30/36) showed either improvement (1/36; 2.7%) or resolution (29/36; 80.6%).

**Conclusions:**

We observed a trend towards normalization of most epileptiform patterns except LPDs and LRDA, over time in patients with acute brain injury. The clinical significance of this trend as it relates to antiseizure medication treatment and neurologic outcomes warrants further investigation in an independent cohort.

## Introduction

A seizure occurring in close temporal relation to a brain insult is considered an acute symptomatic seizure, which is a recognized complication of acute brain injury [1, 2]. Patients with acute symptomatic seizures are at risk for secondary brain injury, development of epilepsy, and worse outcomes [3–6]. Therefore, acute symptomatic seizures typically prompt initiation of an antiseizure medication (ASM) and patients with acute brain injury are often prescribed ASMs as seizure prophylaxis [7–9].

The International League Against Epilepsy’s 2010 definition of acute symptomatic seizures applies only to those with an unequivocal clinical component. However, the dramatic increase in the use of continuous EEG (cEEG) in critically ill patients, including those with acute brain injury, has led to increased identification of electrographic seizures (ES) and epileptiform patterns [10, 11]. Epileptiform EEG patterns have demonstrated strong associations with seizures [12–14], with certain highly epileptiform patterns such as lateralized periodic discharges (LPDs) during hospitalization for acute brain injury being associated with the development of epilepsy [15, 16]. However, EEG patterns are not static but evolve over time. Routine utilization of cEEG during the acute phase of brain injury provides an opportunity to observe temporal trends of EEG patterns during hospitalization. These trends have not been well described. We recently reported our data on long-term evolution of EEG findings in patients with acute symptomatic seizures followed post-discharge [17]. The current study aims to describe the temporal EEG trends in patients with acute brain injury and acute symptomatic seizures or epileptiform EEG patterns during the acute hospitalization phase. A better understanding of the natural history of electrographic seizures and epileptiform patterns in patients with acute brain injury may inform future studies investigating epileptogenesis, optimal duration of ASM treatment, and functional outcomes.

## Material and Methods

We performed a retrospective chart review of all patients undergoing cEEG monitoring between January 1st and June 30th, 2019, at two large academic centers (Yale New Haven Hospital and Cleveland Clinic, Ohio). Patients aged ≥ 18 years with *all* the following criteria were included: a determined etiology of acute brain injury, at least one electrographic seizure or epileptiform abnormality including rhythmic and periodic patterns and spontaneous epileptiform discharges but excluding generalized rhythmic delta activity (GRDA) on day one of cEEG, and at least two calendar days of EEG recording. The etiologies of acute brain injury included were ischemic stroke (IS), intracranial hemorrhage (intraparenchymal (IPH), subdural (SDH), subarachnoid (SAH)), traumatic brain injury (TBI), recent neurological surgery, central nervous system (CNS) infection, or other acute neurological injury. Brain injury categories were not mutually exclusive (e.g., a patient with traumatic brain injury and SAH and IPH would be counted in all three categories). Exclusion criteria were as follows: primary etiology of toxic-metabolic encephalopathy or systemic illnesses (since often reversible), malignant brain tumors (since progressive), hypoxic-ischemic brain injury following cardiac arrest (a unique subpopulation warranting independent study), and those with a history of epilepsy (as EEG findings may be chronic/pre-existing).

Patient demographics, hospitalization details, clinical factors, EEG findings, ASM use, and outpatient follow-up were ascertained by electronic health record review. Data on seizures and epileptiform patterns were collected from EEG reports. EEGs at both institutions were interpreted by board certified epileptologists or clinical neurophysiologists. Variables were analyzed using the Fisher’s exact test, the Chi-square test, or the Mann–Whitney test, as appropriate. P values less than 0.05 were considered significant. The study was approved by the Institutional Review Board of both sites. Informed consent was not required for this observational study. The study is reported in accordance with the Strengthening the Reporting of Observational Studies in Epidemiology (STROBE) guidelines [18].

To visualize the temporal evolution of cEEG patterns, we assigned a numerical score to each pattern/finding. We considered electrographic status epilepticus (ESE) to be the most pathologic finding followed by electrographic seizure (ES). In this study of neurophysiologic trends, we document seizures detected by EEG as electrographic seizures and did not capture if a clinical correlate was described. The remaining patterns were rank ordered by their associations with seizure [11]: ESE = 7, ES = 6, brief potentially ictal rhythmic discharges (BIRDs) = 5, lateralized periodic discharges (LPDs) = 4, lateralized rhythmic delta activity (LRDA) = 3, generalized periodic discharges (GPDs) = 2, sporadic epileptiform discharges (SEDs) = 1, no epileptiform finding = 0. Each day of cEEG was assigned a numerical value according to the most pathologic abnormality documented in the EEG report on that day. The assigned daily values were plotted as a heat map to visualize temporal trends. Analyses were performed using GraphPad Prism 10. The classification of cEEG findings evolution was as follows: *resolution*, indicating a score of 0 (i.e. no epileptiform abnormality) on the final day of monitoring; *improvement*, indicating a decrease in score (using the rank-order scale above) showing a transition to a potentially less epileptogenic pattern from the first day to the last monitoring day; *worsening*, indicating an increase in score (using the rank-order scale above) signifying a shift to a potentially more epileptogenic pattern from the first day to the last day; or *persistent*, indicating that there was no change in score from the first day to the last day.

## Results

The study cohort selection is shown as a flowchart in Supplementary Fig. 1. One hundred and one patients met *all the* inclusion criteria. A total of 535 calendar days of EEG reports were reviewed.

### Study Cohort

The median age of the study cohort was 64 (inter-quartile range (IQR) = 55–74) years and included 64 (63.4%) women (Table 1). Clinical acute symptomatic seizure was reported in 30/101 patients (29.7%) prior to the start of cEEG. The distribution of acute brain injury etiology was: IPH (37/101; 36.6%), IS (31/101; 30.7%), SAH (23/101; 22.8%), SDH (17/101; 16.8%), TBI (13/101; 12.9%), other (16/101; 15.8%), and CNS infection (9/101; 8.9%). Five patients (5.0%) had SAH and IS, 4/101 (4.0%) had IS and IPH, 9/101 (8.9%) had TBI and IPH (with 7 of these patients having TBI, IPH and SAH and six of these having TBI, IPH, SAH, and SDH).

### Patterns and Evolution

**Figure 1:**
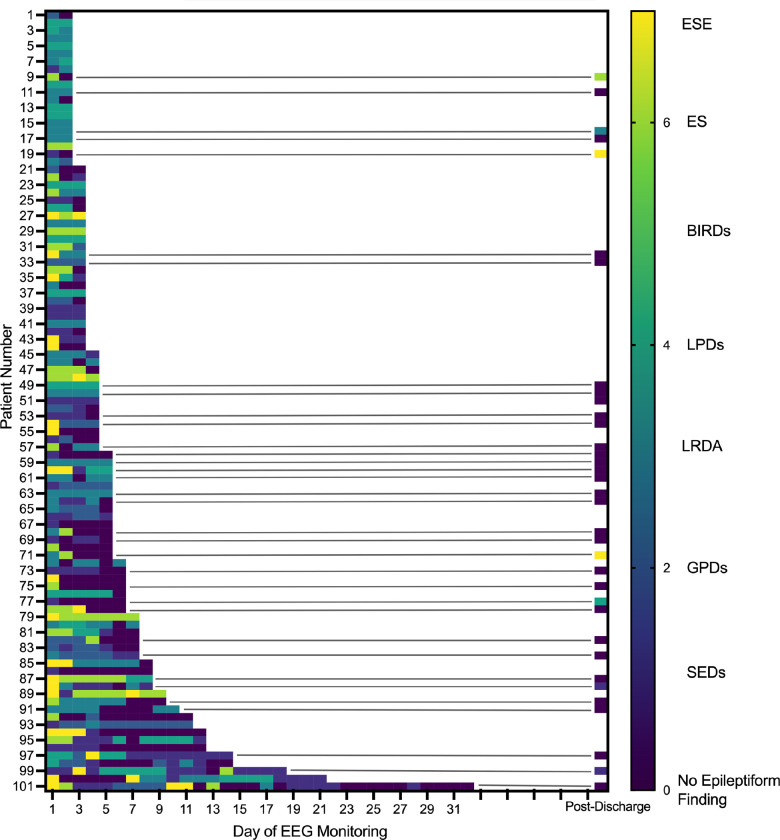
EEG Patterns in Patients with Acute Brain Injury. A heat map displays the most epileptic pattern present on each day of CEEG for each patient. An asterisk signifies that a patient’s pattern persisted or worsened from the first day to the last day of CEEG. ESE = Electrographic Status Epilepticus, ES = Electrographic Seizure, LPDs = Lateralized Periodic Discharges, LRDA = Lateralized Rhythmic Delta Activity, GPDs = Generalized Periodic Discharges, SEDs = Sporadic Epileptiform Discharges.

**Figure 2:**
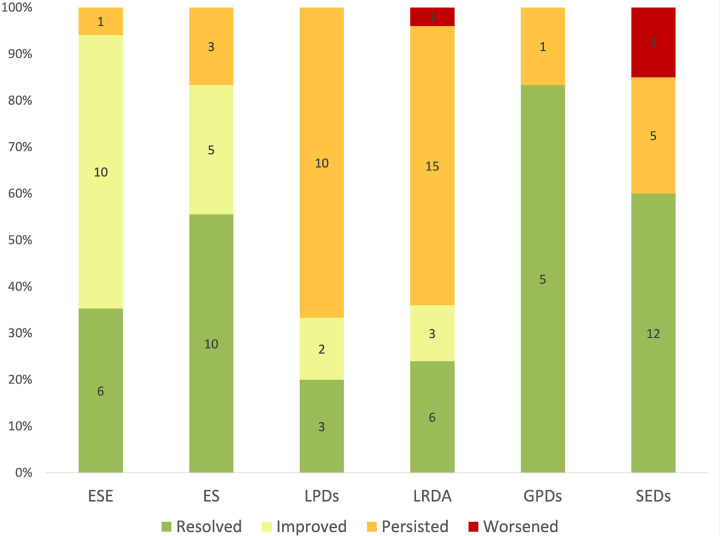
Evolution of EEG Patterns During Hospitalization for Acute Brain Injury. A stacked bar chart shows the percentages of patients with each category of initial EEG finding that had resolution, improvement, persistence, or worsening of pattern score between the first and the last day of in-hospital monitoring. ESE = Electrographic Status Epilepticus, ES = Electrographic Seizure, LPDs = Lateralized Periodic Discharges, LRDA = Lateralized Rhythmic Delta Activity, GPDs = Generalized Periodic Discharges, SEDs = Sporadic Epileptiform Discharges.

The median duration of cEEG was 4 (IQR 3–6) days. Twenty-three patients had seven or greater EEG days for review. Figure 1 shows the daily evolution of EEG findings at the individual patient level charted as a heat map. Fifty-eight patients had seven or more calendar days elapse between their date of admission and the final cEEG epoch during hospitalization. Patients with ESE or ES on day one of cEEG were monitored longer (5 days (IQR 3–8) vs. 4 (IQR 2–5), p = 0.01) compared to those without. Table S1 delineates the distribution of days of cEEG monitoring by initial EEG pattern. Figure 2 shows group level EEG transitions during hospitalization. The worst initial cEEG pattern was ESE in 17/101 (16.8%) patients, ES in 18/101 (17.8%), LPDs in 15/101 (14.9%), LRDA in 25/101 (24.8%), GPDs in 6/101 (5.9%), and SEDs in 20/101 (19.8%). No patients had BIRDs as their most pathologic initial EEG finding. In patients with ESE on the first day of monitoring, 6/17 (35.3%) had resolution of epileptiform patterns by the final day of monitoring, 10/17 (58.8%) transitioned to a less epileptiform pattern representing improvement, and 1/17 (5.9%) had persistence of ESE. In patients with ES, 10/18 (55.5%) had resolution, 5/18 (27.8%) had improvement, and 3/18 (17.6%) had persistence. LPDs seen on day one persisted unchanged in 10/15 (66.7%) patients and improved or resolved in 5/15 (33.3%); none worsened by transitioning to a more epileptiform pattern/finding. LRDA persisted unchanged in 15/25 (60.0%) patients, improved or resolved in 9/25 (36.0%), and worsened in 1/25 (4.0%). GPDs persisted in 1/6 (16.7%) patients and resolved in 5/6 (83.3%). For patients with SEDs, 12/20 (60.0%) resolved, 3/20 (15.0%) worsened, and 5/20 (25.0%) persisted. Overall, 62 patients (61.4%) had either improvement or resolution, with a breakdown of 41.6% fully resolved and 19.8% improved.

### ASM utilization

In this cohort, 95/101 (94.1%) patients were treated with ASMs. Thirty-eight patients (37.6%) were started on more than one ASM and 12/101 (11.9%) were prescribed three or more ASMs. All 35 patients with ESE or ES were prescribed least one ASM. Among the patients without ESE or ES, 60/66 (91%) were prescribed at least one ASM. Patients with ESE or ES were more frequently on multiple ASMs compared to those with rhythmic/periodic patterns (26/35 (74.3%) vs. 12/60 (20%), p < 0.001, OR 12 (95% CI 4–31)). Of the 95 patients prescribed ASMs, 89 (93.7%) received levetiracetam, 28 (29.5%) lacosamide, 12 (12.6%) fosphenytoin/phenytoin, and 7 (7.4%) valproic acid.

### Outcomes and Post-Hospitalization Data

**Figure 3:**
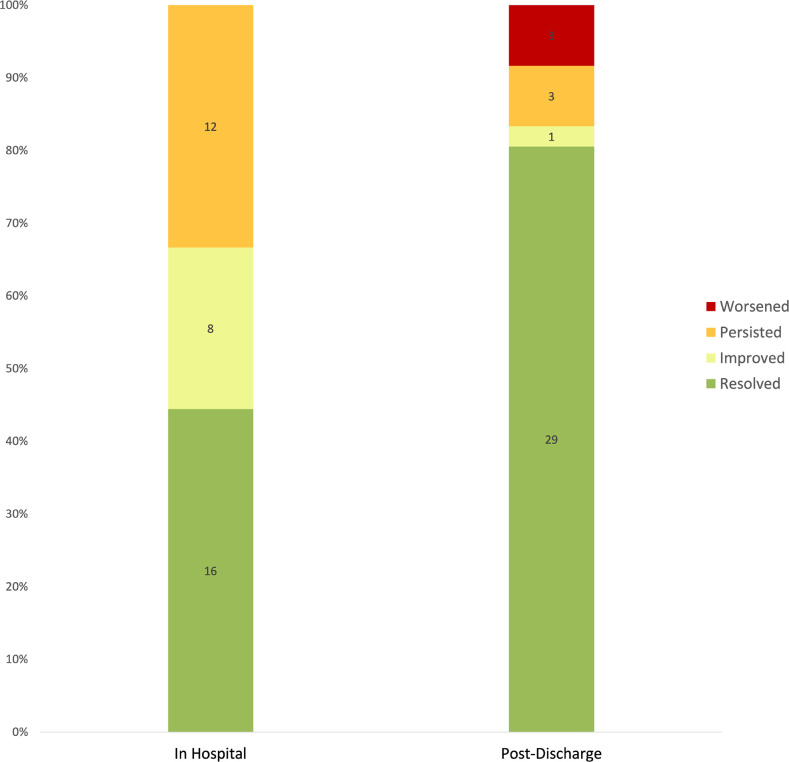
Long-term evolution of EEG Patterns on Post-Discharge EEG: A stacked bar chart shows the percentages of patients who had resolution, improvement, persistence, or worsening of EEG finding score between the first day of monitoring and the final day in during hospitalization (In Hospital Group) and the first day of monitoring (during hospitalization) and on the first post-discharge EEG.

Twenty patients in this cohort died during the initial inpatient hospitalization phase of acute brain injury (20/101; 19.8%). Among 81 survivors, 61 (75.3%) were discharged on ASMs, and 15 (18.5%) had a clinical seizure after discharge (13/15 patients (86.7%) on ASMs). Post-discharge EEGs were available for 36/81 (44.4%) patients of which 15/36 (41.7%) were performed in a subsequent inpatient encounter. Among them, 29/36 (80.5%) were free from seizures or epileptiform patterns (Fig. 3). Only 16/36 (44.4%) had complete resolution of epileptiform patterns/discharges during the initial hospitalization. The median time from hospital discharge to post-hospitalization EEG was 143 (IQR = 81.5–231.8) days (4.5 months; IQR 3–8).

## Discussion

We describe the temporal evolution of epileptiform EEG patterns in a cohort of patients with acute brain injury. We observed a resolution or improvement in ESE and ES in most patients, possibly attributable to more aggressive treatment. Our findings align with a recent international survey of neurologists, which showed that over 95% of respondents treat clinical or electrographic acute symptomatic seizures [7]. Additionally, patients in our study who experienced ESE and ES received more days of monitoring and were more likely to be on multiple ASMs, suggesting that clinicians continued EEG monitoring to evaluate for improvement. Lateralized patterns (LPDs and LRDA) more frequently persisted until the final day of cEEG. Several reasons may account for this. It is plausible that LPDs or LRDA result from structural injuries more associated with persistent epileptic potential. A cohort study of patients undergoing cEEG during acute illness found that patients with LPDs were seven times more likely to develop epilepsy than those with nonperiodic or nonepileptic patterns. [15]. It is also likely that patients were disconnected from EEG while these patterns persisted if clinicians deemed cEEG data was no longer necessary to guide treatment. Conversely, GPDs and SEDs more frequently improved or resolved, yet the reasons remain unclear. It is possible that these patterns arise from concomitant toxic-metabolic processes that resulted in only transient cortical irritability or from less severe structural cortical brain injury. To identify a sample with a standardized and adequate period of EEG monitoring, we included only patients with EEG data from two separate calendar days. This however presents the limitation that rapidly improving patterns that resolved on calendar day one may have resulted in discontinuation of EEG and therefore exclusion from our cohort. Further investigations are needed to delineate the cerebral pathophysiology associated with epileptiform abnormalities in the setting of acute brain injury. Further study is also needed to better understand optimal ASM use and how this may be guided by pattern recognition or resolution.

In patients with available follow-up, 15 had seizures after discharge (18.5% of patients alive at discharge), which is comparable to a previous cohort of patients undergoing cEEG [15]. Most surviving patients were discharged on ASMs, aligning with international practice patterns identified in a recent survey [7]. In the small subgroup of patients with available post-discharge EEGs, most epileptiform patterns showed improvement or resolution consistent with prior studies [17, 19]. A greater proportion of patients had complete resolution of epileptiform patterns on follow-up than during the acute hospitalization phase. This suggests that evolution of EEG patterns continues beyond the acute hospitalization period in brain injured patients. A small study of ischemic stroke survivors who had acute symptomatic seizures during hospitalization showed the prognostic benefit of outpatient EEG whereby the presence of epileptiform abnormalities was significantly correlated with epileptogenesis [20]. Similarly, a recent publication from our group found that most patients have resolution of epileptiform EEG findings on outpatient follow-up and that the persistence of epileptiform abnormalities on follow up EEG was associated with seizure recurrence [17].

Post-acute symptomatic seizure (PASS) clinics with outpatient EEGs represent an emerging approach to follow-up for patients with acute symptomatic seizures and acute epileptiform EEG patterns post-discharge. PASS clinics improve access to specialty care and provide an opportunity to identify patients who no longer need ASMs and also those who are at highest risk for developing epilepsy [17, 21].

## Strengths and Limitations

Our study presents a thorough investigation of the evolution of epileptiform EEG abnormalities in patients with acute brain injury at two large academic centers. With a two-center approach, our findings encourage generalizability to institutions with similar infrastructure. Our study extends the period of monitoring well beyond previous studies that focused on EEG patterns only within the first 72 hours of monitoring [22]. With a median of four cEEG days and more than half of patients with EEG data exceeding seven days following admission, we observed the temporal evolution of patterns throughout the monitored portion of the initial hospitalization following an acute brain injury.

Our study has several important limitations. It is retrospective and relies on EEG data collected for clinical decision-making rather than for the purpose of evaluating the natural history of EEG findings. Furthermore, patients in this study had no systematic follow-up, limiting our ability to interpret our follow-up EEG data and to assess the rate of seizure recurrence after discharge. Both centers are tertiary care centers performing cEEG in a large volume of patients, potentially limiting generalizability. We used a rating system for EEG findings based on known associations with the occurrence of seizures [12]. While our rating system was ordinal in nature, EEG patterns are not necessarily expected to transition directly across this numerical gradient. Finally, while our cohort was relatively large for a study of EEG patterns in acute brain injury derived from a 6-month period, the absolute numbers of patients in disease specific subgroups are small and therefore limited our ability to compare groups.

## Conclusions

This study describes the in-hospital evolution of EEG patterns for patients with acute brain injury. Nearly two-thirds of patients demonstrated improvement or resolution in their initial EEG findings prior to discharge, which further improved to > 80% when a follow up EEG was done. LPDs and LRDA tend to persist beyond the acute hospitalization phase, perhaps reflective of the degree of structural injury. Insights gained from studying the evolution of EEG patterns may enhance our understanding of the significance of acute symptomatic seizures and epileptogenic patterns in acute brain injury. Future multicenter studies with larger sample sizes are needed to validate these findings, particularly in subgroups, and elucidate the broader clinical implications for patient management and prognosis.

## Figures and Tables

**Table 1:** Demographics, Clinical Characteristics, and Outcomes in this Cohort of Patients with Acute Brain Injury and Seizures or Epileptiform EEG Findings

Variable, Total Cohort (n=101)	Number (%) or Median (IQR)
**Demographics**	
Female	64 (63.4%)
Age	64 (55–74)
	
**Clinical Characteristics**	
Intubated prior to EEG	43 (42.6%)
GCS at time of EEG monitoring initiation	9 (7–13)
Clinical seizure prior to EEG	30 (29.7%)
	
**Etiology of Brain Injury**	
Acute ischemic stroke	31 (30.7%)
Subarachnoid hemorrhage	23 (22.8%)
Intraparenchymal hemorrhage	37 (36.6%)
Subdural hematoma	17 (16.8%)
Traumatic brain injury	13 (12.9%)
CNS infection	9 (8.9%)
Other	16 (15.8%)
	
**Initial Electrographic Findings**	
Status Epilepticus	17 (16.8%)
Seizure	18 (17.8%)
LPDs/BiPDs	15 (14.9%)
LRDA	25 (24.8%)
GPDs	6 (5.9%)
SEDs	20 (19.8%)
	
**Antiseizure Medication Treatment**	
One medication	57 (56.4%)
Two medications	26 (25.7%)
Three medications	8 (7.9%)
Four medications	2 (2.0%%)
Five medications	0 (0.0%)
Six medications	1 (1.0%)
Seven medications	1 (1.0%)
Anesthesia for seizure control	8 (7.9%)
	
**Antiseizure Medications Used** ***	
Levetiracetam	89 (93.7%)
Fosphenytoin/phenytoin	12 (12.6%)
Valproic acid	7 (7.4%)
Lacosamide	28 (29.5%)
Zonisamide	5 (5.3%)
Other	13 (13.7%)
	
**Outcomes**	
Length of stay	16 (10–29)
GOS at discharge	3 (3–4)
Died in hospital	20 (19.8%)
Total days of EEG monitoring	4 (3–6)
Days from admission to final EEG	8 (4–14)
Discharged on antiseizure medication*	61/81 (75.3%)
Seizure after discharge	15 (14.9%)
Days from discharge to follow-up EEG	143 (81.5–231.8)
Months from discharge to last follow-up*	31 (7–47)

IQR = Inter-quartile range

* Among the 81 survivors at discharge.

** Not mutually exclusive; patients could have more than one etiology.

*** Among the 95 patients treated with antiseizure medications.
